# Left Septal Fascicular Block Following Left Bundle Branch Area Pacing

**DOI:** 10.19102/icrm.2025.16044

**Published:** 2025-04-15

**Authors:** Ahmet Lutfu Sertdemir, Ahmet Taha Sahin, Andrés Ricardo Pérez-Riera, Enes Elvin Gul, Adrian Baranchuk

**Affiliations:** 1 Department of Cardiology, Necmettin Erbakan University School of Medicine, Konya, Turkey; 2 Department of Cardiology, Beyhekim Training and Research Hospital, Konya, Turkey; 3 Design of Studies and Scientific Writing Laboratory, ABC School of Medicine, Santo André, São Paulo, Brazil; 4 Division of Cardiology, Kingston Health Science Center, Queen’s University, Kingston, Ontario, Canada

**Keywords:** Case report, conduction system pacing, left bundle branch area pacing, left septal fascicular block

## Abstract

Left bundle branch area pacing (LBBAP) is a type of conduction system pacing wherein the left bundle branch and/or the left side of the interventricular septum are stimulated with a permanent pacing lead to maintain physiological electrical activation of the left ventricle. As understanding grows regarding trifascicular activation in the left ventricle and left septal fascicular block (LSFB), there is an indication that new electrocardiographic alterations may emerge, particularly in instances of arterial occlusions. Here, we present a case study delineating LSFB subsequent to LBBAP.

## Case presentation

**Figure 1:**
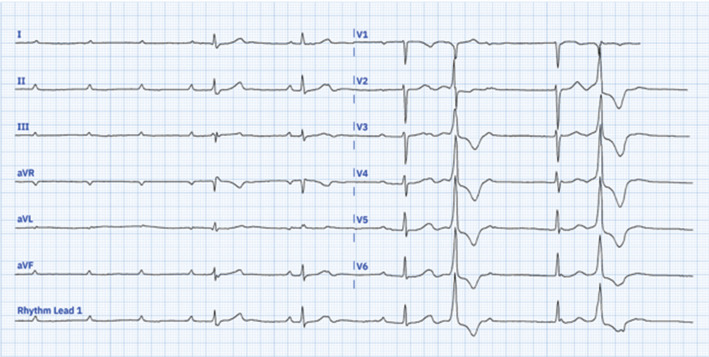
Admission 12-lead electrocardiogram showing advanced atrioventricular block with narrow escape beats and premature ventricular complexes.

**Figure 2:**
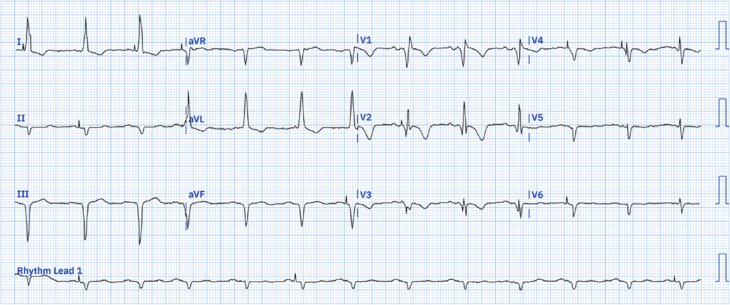
Electrocardiogram obtained after the procedure. Electrocardiogram diagnosis: P-wave duration, 200 ms; terminal mode negative of P-wave in V1 > 40 ms; Morris index > 0.04 mm/s; QRS axis, −46°; R-wave peak time in aVL ≥ 45 ms; QIII > QII: inferior infarction + left anterior fascicular block; QRS duration, 108 ms; increased ventricular activation time in V1 and V2 ≥ 35 ms: left septal fascicular block (LSFB). Conclusion: left atrial enlargement + transmural inferior myocardial infarction + left anterior fascicular block + left septal fascicular block + left ventricular hypertrophy (strain pattern of repolarization in the lateral leads) + pacemaker spikes.

**Figure 3:**
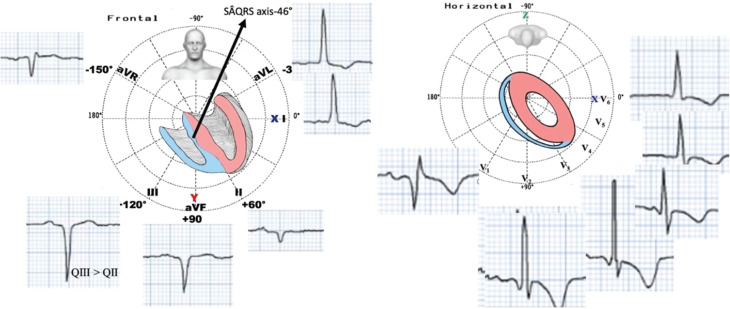
Typical electrocardiogram/vectorcardiogram of left septal fascicular block in the frontal and horizontal planes.

A 36-year-old female patient with a history of congenital atrioventricular block (AVB) presented to the emergency room with lightheadedness and presyncope. Upon admission, her heart rate was recorded at 42 bpm, and her blood pressure measured 96/58 mmHg. The physical examination was unremarkable. Laboratory analyses yielded results within normal ranges. An admission 12-lead electrocardiogram (ECG) revealed advanced AVB, junctional escape rhythm at 40 bpm, and frequent unifocal premature ventricular complexes **(Figure 1)**. Transthoracic echocardiography revealed preserved left ventricular systolic and diastolic functions. Due to symptoms and the ECG evidence of advanced AVB, she was scheduled for a permanent pacemaker implantation. She underwent a successful left bundle branch area pacing (LBBAP) procedure. Post-procedural ECG exhibited sinus rhythm, extreme left QRS axis deviation (SÂQRS ≈ −45°: left anterior fascicular block [LAFB]), normal QRS duration, pacemaker spikes, disappearance of the initial Q-waves in I and aVL, and prominent anterior QRS forces in the right precordial V1–V2 leads caused by left septal fascicular block (LSFB) **(Figures 2 and 3)**. No further complications were observed during the patient’s follow-up, leading to her discharge.

## Discussion

Here, we describe a case of LSFB following LBBAP. This phenomenon has not been well investigated before. To our knowledge, there is only one recent report of LSFB unmasked by left fascicular pacing.^1^

LBBAP is a promising physiological pacing modality shown to promote better electrical and mechanical synchrony, which was introduced by Vijayaraman et al. in 2019.^2–4^ Successful LBBAP is characterized by a unipolar paced QRS complex exhibiting a morphology resembling right bundle branch block (RBBB), with a duration of ≤130 ms.^5^

Trifascicular activation of the left ventricle is now widely accepted by researchers.^6^ The most important feature of LSFB, as included in its diagnostic criteria, is that it tends to be intermittent.^7^ It has also been proven that this phenomenon is associated with phase 4 bradycardia and its relationship with early atrial extra stimulus.^8^ There are studies showing that the main cause of LSFB is severe stenosis before the first septal perforator branch of the left anterior descending (LAD) artery.^9,10^

The ECG criteria for LSFB have been described previously. The criteria currently proposed are shown in **Table 1**. The transient prominent anterior QRS forces in V2–V3 and the disappearance of the initial Q-wave in I and aVL on ECG-2 (indicating the absence of the first vector of ventricular activation due to LSFB) reinforce the diagnosis of this dromotropic disturbance.^10^ Our case fulfilled most of the ECG criteria of LSFB.

**Table 1:** Diagnostic Criteria of Left Septal Fascicular Block

LSFB Criteria	Present in this Case?
Presence of PAF of QRS	Yes
Normal QRS duration or discrete increase when not associated with other blocks	Yes
Prolonged R-wave peak time in aVL ≥ 40 ms	Yes
R-wave voltage in V1 ≥ 5 mm	Yes
R/S ratio in V1 > 2	Yes
Possible small Q-wave in V2 and V3 or V1 and V2	Yes

*Abbreviations:* LSFB, left septal fascicular block; PAF, prominent anterior force.

According to authors in this field, the transient appearance of LSFB accompanied by significant anterior forces may serve as a potential indicator of impending acute coronary syndrome.^10,11^ We previously encountered a case where we suspected critical occlusion of the LAD coronary artery before the first septal perforator branch, arising as a complication subsequent to alcohol septal ablation in a patient with hypertrophic cardiomyopathy.^12^ Pérez-Riera et al. proposed that the temporary occurrence of LAFB and LSFB following self-expandable percutaneous transcatheter aortic valve implantation could be attributed to septal ischemia and fibrosis.^13^ In our case, the mechanism of LSFB following LBBAP can be explained by the capture of the left posterior fascicles. In the current case, LSFB was accompanied by a typical LAFB and complete RBBB with a left axis deviation.

In conclusion, the transient emergence of LSFB accompanied by notable anterior forces may warrant attention as a potential indicator of septal ischemia and/or fibrosis following LBBAP.
